# Speed-accuracy tradeoffs influence the main sequence of saccadic eye movements

**DOI:** 10.1038/s41598-022-09029-8

**Published:** 2022-03-28

**Authors:** Leslie Guadron, A. John van Opstal, Jeroen Goossens

**Affiliations:** 1 Department of Cognitive Neuroscience, Donders Institute for Brain, Cognition and Behaviour, Radboudumc, P.O. Box 9101, 6500 HB Nijmegen, The Netherlands; 2 Department of Biophysics, Donders Institute for Brain, Cognition and Behaviour, Radboud University, P.O. Box 9010//066, 6500 GL Nijmegen, The Netherlands

**Keywords:** Saccades, Superior colliculus, Motor control, Oculomotor system, Visual system, Neuroscience, Perception

## Abstract

Several studies have proposed that an optimal speed-accuracy tradeoff underlies the stereotyped relationship between amplitude, duration and peak velocity of saccades (main sequence). To test this theory, we asked 8 participants to make saccades to Gaussian-blurred spots and manipulated the task’s accuracy constraints by varying target size (1, 3, and 5°). The largest targets indeed yielded more endpoint scatter (and lower gains) than the smallest targets, although this effect subsided with target eccentricity. The main sequence depended on several interacting factors: saccade latency, saccade gain and target size. Early saccades, which were faster than amplitude-matched late saccades, followed the target-size dependency one would expect from a speed-accuracy tradeoff process. They had higher peak velocities and shorter durations for larger targets than for smaller targets. For late saccades, however, the opposite was found. Deviations from the main sequence also covaried with saccade gain, in line with the idea that motor noise underlies part of the endpoint variability. Thus, our data provide partial evidence that the saccadic system weighs the detrimental effects of motor noise on saccade accuracy against movement duration and speed, but other factors also modulate the kinematics. We discuss the possible involvement of parallel saccade pathways to account for our findings.

## Introduction

Saccades are fast eye movements made to foveate different parts of the visual field. Their trajectories are highly stereotyped as quantified by the so-called saccade main-sequence, which describes the tight relationships between the saccade amplitude and duration, and between the amplitude and peak velocity^1,2^. These relationships are generally conserved from one individual to another and it has been hypothesized that they betray a neural strategy of the saccadic system that aims to optimize speed and accuracy^3,4^.

There are circumstances under which saccades can deviate from the main sequence, which is generally valid for reflexive, visually guided saccades. For example, memory-guided saccades^5^ and antisaccades^6^ both lead to slower movements than normal, visually-evoked saccades of the same amplitude. Cognitive influences in saccade tasks can also lead to deviations from the main sequence. For example, saccades made to rewarding visual stimuli, like faces, are typically faster than saccades made to meaningless target spots^7,8^.

It is not yet fully understood which factors contribute to the optimization of saccadic eye movements. It has been proposed that accuracy and speed are crucial when planning an eye movement. A saccade should be quick because during the movement visual input is partly suppressed^9^. Thus, the sooner the eye movement ends, the sooner high-acuity vision is regained. However, a movement that is too quick will not be very accurate due to the signal-dependent noise accompanying the control signal^10^. The best eye movement can therefore be said to be one that minimizes duration while maintaining as much accuracy as possible. This is the classic speed-accuracy trade-off that has been well documented in oculomotor behavioral and computational studies^11–14^. It has been shown that this tradeoff can result in the main-sequence relationships^3^.

Neural models of the saccadic system traditionally assume that the main-sequence properties result from a saturating nonlinearity in the firing rates of saccadic burst cells in the brainstem^15–17^. However, there is surprisingly little evidence for this biophysical account. More recent studies point to the midbrain superior colliculus (SC) as a possible source for the nonlinear main sequence^4,18,19^. Because the SC is an important sensorimotor interface in the control of saccade behavior^20–22^, it is in an ideal position to optimize the speed-accuracy trade-off.

In this paper, we aimed to test the hypothesis that the principle of minimizing the consequences of motor noise in a speed-accuracy trade-off underlies the main sequence relationships. We reasoned that visual targets of different sizes would impose different accuracy constraints on saccades. Larger targets would permit less accurate saccades than smaller targets, which would allow for higher peak velocities and shorter durations. Conversely, because of the higher precision demands, we expected lower peak velocities and longer durations for saccades to smaller targets. Thus, if a speed-accuracy trade-off underlies the main sequence, we expect that the main-sequence relations change systematically with target size. A previous study has shown that target size can influence saccade metrics and reaction times^23^. However, the study did not address the effects of target size on the main-sequence relationships.

A complicating factor in testing this theory is that the endpoint variability of saccades to a particular target may be due to a combination of motor noise and target localization noise (e.g., due to noise in the visual system, misperception of the initial eye position, and noise in the SC motor map^4,24^). Smeets and Hooge^25^ realized that localization noise would keep saccades on the main sequence—in our experiments possibly a target-size-specific main sequence. Motor noise, on the other hand, would cause saccades to deviate from the main-sequence relations (which would mask the effect of target size). Indeed, localization noise would only affect the intended amplitude (and direction) of the saccade, but not its execution, whereas motor noise would influence the execution, resulting in a discrepancy between the intended saccade and the actual movement. More specifically, starting from a simple pulse-step model of saccade generation and assuming that saccade duration depends only on the duration (and not the amplitude) of the motor command, Smeets and Hooge predicted that a higher than normal pulse due to noise in the firing intensity of motor neurons would result in a saccade that overshoots its planned endpoint with a higher than normal peak velocity. A lower than normal pulse would result in an undershooting saccade with a lower than normal peak velocity. Thus, if motor noise contributes significantly to the variability in saccade endpoints, this “pulse-height noise hypothesis” predicts that deviations from the (target-size specific) main sequence correlate with the trial to trial variations in saccade gain.

## Methods

### Subjects

Eight healthy subjects (4 male, 4 female) with normal or corrected-to-normal visual acuity participated in the experiments. Their ages ranged from 23 to 50 years. Written informed consent was obtained from each participant before the experiment. The local ethics committee of social sciences at Radboud University (ECSW 2016-2208-41) approved the study before it was carried out and the experimental protocols fully adhered to the Declaration of Helsinki.

Each subject performed between 1540 and 2700 trials. The data were collected in blocks of 270 trials. Subjects were allowed to take a break after every block and the measurements were completed over several sessions.

### Equipment

An EyeLink 1000 Plus (SR research) was used to collect binocular eye position and gaze data at a sampling rate of 500 Hz. The eye gaze data were collected using a laptop computer. The experimental stimulus software was written in MATLAB 2014 using the Psychophysics Toolbox extension^21,22^ and executed on a desktop computer equipped with an open GL graphics card. A 32-in. screen (Dell UP3214Q) with a refresh rate of 60 Hz and a screen resolution of 3840 × 2160 pixels was used to present the stimuli.

### Setup

The experiment took place in a dark room with the only light source being the screen used for target presentation. Subjects were seated 60 cm from the screen and were asked to minimize head movements as much as possible. A chin cup and forehead rest mounted between a pair of vertical posts was used to help stabilize the head. Head movements were also tracked using the EyeLink in Remote mode and placing a tracking sticker on the subject’s forehead. A custom 13-point calibration was carried out prior to the start of the experiment. The calibration points were clustered closer to the horizontal axis of the screen since the subjects would mostly be making horizontal eye movements. The left and right eyes were calibrated separately with the non-viewing eye covered. Otherwise, viewing was binocular and movements of both eyes were recorded.

### The task

The subjects’ task was to make a saccade to the center of the target as quickly and accurately as possible. The instructions for the task were displayed on the screen and subjects were asked to press a button to start the experiment. The subjects then had to maintain fixation on a fixation point at the center of the screen until it disappeared from the screen. The fixation period varied randomly from trial to trial and lasted either 300, 500, 750, 1000 or 1500 ms. If the eyes strayed more than 4° from the center of the fixation point during the fixation period a message was displayed on the screen asking the subject to maintain fixation and the trial was repeated. At the end of the fixation period, the fixation point disappeared and the peripheral target was flashed on the screen for 100 ms (6 frames). Subjects were asked to make their saccade only after the fixation point had disappeared. They had 1 s to complete their saccade. Target presentations were brief since it is now clear that visual signals can be processed during saccades and can even be helpful in guiding behavior^26–28^. We wanted to prevent this because visual feedback could dampen the effect of motor noise during the saccade.

### Stimuli

The targets and fixation point were presented on a gray screen with a luminance of 37.33 cd/m^2^. The fixation point was a white circle with a diameter of 1° and it was presented at the center of the screen. The targets were white blurred spots produced by attenuating the luminance with a 2D Gaussian. The peak luminance was 175 cd/m^2^ and kept constant for the three different targets. We intended to make the boundaries of the targets visually ambiguous so that we could alter the subject’s saccade strategy. The targets’ diameters—6 sigmas of the Gaussian—were 1, 3, and 5°. They were presented at eccentricities of 5, 7, 9, 11, 13, 15, 17, 19, and 21° to the left and right of the fixation point. Trials were presented in pseudorandom order with 3 repetitions of each condition per block.

### Analysis

#### Saccade detection

Saccade onsets and offsets were detected by custom software using separate velocity and acceleration criteria for movement onsets (40°/s, 7500°/s^2^) and offsets (30°/s, − 5000°/s^2^). Movements of the left and right eye were marked independently. All markings were visually inspected and corrected if deemed necessary. Eye velocity was computed from the vector sum of the horizontal and vertical eye velocity components. Eye acceleration was the time derivative of this eye velocity signal. Rare occurrences of saccades with latencies less than 100 ms were discarded from all analyses to make sure all included saccades were executed in complete darkness.

#### Saccade metrics

The variability in saccade endpoints was quantified by bivariate contour ellipse areas (BCEA) for each target eccentricity and each target size. The ellipse area covering 1 standard deviation of a bi-variate Gaussian distribution was obtained from the following equation:

1 $$A=2 k \pi {\sigma }_{H}{\sigma }_{V} {(1-{\rho }^{2}) }^{1/2}$$

where *A* is the area of the ellipse, ${\sigma }_{H}$ and ${\sigma }_{V}$ are the standard deviations along the horizontal and vertical meridians, and $\rho$ is the product-moment correlation of the two position components^29^. The value $k$ was set to 1.14, yielding 68.3% probability that a given observation falls within the elliptical area.

The gain was defined as the saccade amplitude divided by the actual target eccentricity on the retina as computed from the target location on the screen minus the initial gaze position of the eye. In this way, saccades overshooting the target center had a gain > 1 and saccades undershooting the target center a gain < 1.

We used (generalized) linear mixed-effects regression models to test if there were any statistically significant effects of target eccentricity and target size on the BCEA, and saccade gain (Matlab function fitlme) and on the number of late saccades (using fitglm with a binomial distribution). These models were fit using maximum pseudo likelihood estimation. We allowed for random effects on slope and offset. The random effects were grouped by subject, by movement direction within subject, and by recorded eye within movement direction to accommodate the nested structure of the repeated measurements.

#### Saccade kinematics

To assess the effects of target size, saccade latency and saccade gain on the main sequence relations, we first fitted the amplitude—duration and amplitude—peak velocity relationships for saccades of each individual subject. We assumed an affine relationship between saccade amplitude, *R* (in deg), and saccade duration, *D* (in ms):

2 $$ D = a*R + b $$

with constants *a* in ms/deg and *b* in ms. In addition, we assume a tight linear relation between saccade amplitude and the product of peak velocity and duration:

3 $$ Vp*D = k*R $$

Combining Eqs. (2) and (3)^30^ leads to the nonlinear relation between saccade amplitude and saccade peak velocity, *Vp* (in °/s):

4 $$ Vp  = 1/\left( {\alpha /R + \beta } \right) $$

with α = *b/k* and β = a/k. *Vp* saturates for *R* → ∞ with a value *Vp*_*max*_ = 1/β (in °/s), and decreases for *R* → 0 with a slope of 1/α (in ms^-1^). Only saccades with latencies < 250 ms were included in these fits and data were stratified by target size, saccade direction and recorded eye. Parameters *a* and *b* were estimated with standard linear regression (Matlab function fitlm). To estimate *α* and *β* we fitted 1/*Vp* as an affine function of 1/*R* using general linear modeling with a reciprocal link function (using fitglm).

Adopting the main sequence functions obtained for “stereotyped” saccades to the smallest-diameter targets as personalized reference, we then computed normalized saccade durations and normalized saccade peak velocities for all saccades:

5 $$ D_{normalized} = \, D_{measured} /D_{predicted} \left( {R_{measured} } \right) $$

6 $$ Vp_{normalized} = \, Vp_{measured} /Vp_{predicted} \left( {R_{measured} } \right) $$

where *D*_*predicted*_*(R*_*measured*_*)* is the predicted duration given the measured amplitude of the saccade, *R*_*measured*_, and *D*_*measured*_ is the measured duration of the saccade. Likewise, *Vp*_*predicted*_*(R*_*measured*_*)* is the peak velocity predicted for a given saccade based on its amplitude, *R*_*measured*_, and *Vp*_*measured*_ is the measured peak velocity of that saccade. This normalization allowed for pooling of the kinematics data across saccade amplitude. “Stereotyped'' saccades included in the main sequence fits that define the denominators were those saccades to 1° wide targets that had single-peaked velocity profiles, latencies < 250 ms, except for the 5% shortest and longest latencies, and gains falling within the central 50% of the cumulative gain distribution. In addition to these individualized inclusion criteria, we used separate normalizing main sequence fits for each eye and each saccade direction.

The normalized duration and peak velocity data were then plotted as a function of either saccade latency or saccade gain and analysed with mixed-effects linear regression models (using fitlme). In these models, fixed effects were either latency and target size or gain and target size. We performed these analyses first on data from each individual subject (grouping random effects by eye and saccade direction) and then on data pooled across subjects (adding subject as random-effect grouping variable).

Finally, we modeled saccade duration (using fitlme) and saccade peak velocity (using fitglme) directly with fixed effects for saccade amplitude [according to Eqs. (2) and (3)], target size, saccade latency, saccade gain and their interactions. Latency data from each participant were first centered on the participant’s median reaction time. Random effects on the slopes and offsets were again grouped by subject, by movement direction within subject, and by recorded eye within movement direction. Interaction terms that did not improve the models statistically (likelihood ratio test using compare), were dropped in a stepwise, backward model selection procedure. 

Terms included in the different mixed-effects regression models can be found in the corresponding tables of the Supplementary Materials along with the relevant statistics. Fixed effects with a P-value less than 0.05 (Type I error) were considered statistically significant.

## Results

### Endpoints

**Figure 1:**
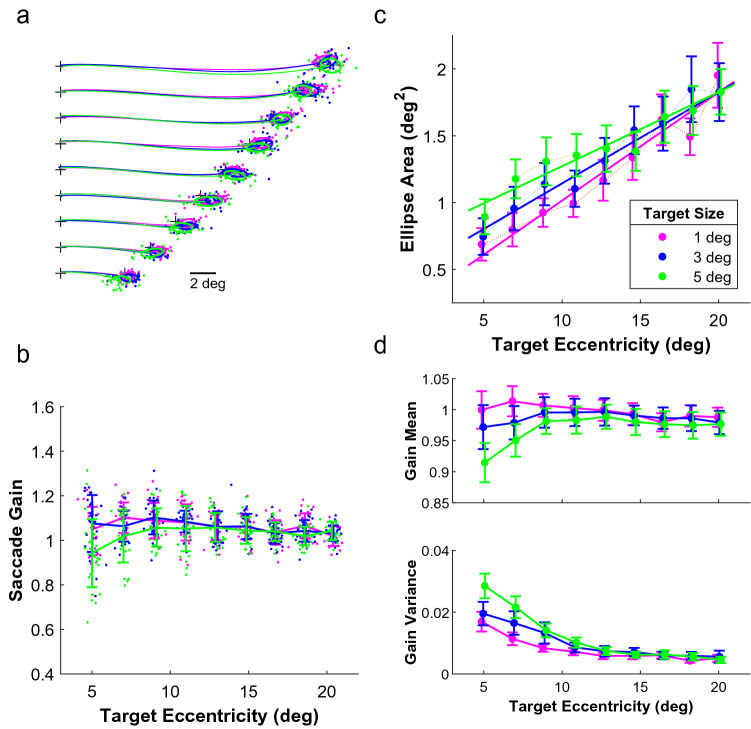
Endpoint variability. (**a**) Individual dots show the endpoints of the saccades made to the different target locations and different target sizes (color coded) by one of our subjects. The lines represent the average saccade trajectories from the fixation point (+) to the peripheral targets. The ellipses represent the distributions of the endpoints. Only rightward saccades of the subject’s right eye are shown. (**b**) Amplitude gain of the same rightward saccades as a function of target eccentricity. A gain of 1 corresponds to a saccade that lands at the center of the briefly (100 ms) flashed target. Solid lines indicate the mean, error bars indicate ± 1 standard deviation. (**c**) Effect of target eccentricity and target size on endpoint variability. Larger ellipse areas reflect more scatter among the endpoints. The solid lines are the result of a mixed effects model fitted to all data (both eyes and both target directions) from all subjects. Dashed lines are grand averages from all 8 subjects. Error bars indicate ± 1 standard deviation of the grand means. (**d**) Grand mean of the saccade gain and the grand mean of the gain variance for different target eccentricities. Error bars indicate ± 1 standard deviation of the grand means.

Figure 1a summarizes our subjects’ task. They made horizontal saccades to targets of three different sizes (color coded) presented at nine different eccentricities. Targets were flashed briefly (100 ms) to ensure that no visual information was available during the saccades. The averaged two-dimensional trajectories and the individual endpoints shown here were obtained from the right eye of one of our participants.

Figure 1b shows the gain of these rightward saccades made to the three different target sizes at different target eccentricities. Individual points represent individual saccades of the participant's right eye. Averaged across subjects (and eyes and saccade direction), the difference in gain between the target sizes is largest at the smallest target eccentricities. Saccades made to the 5° target appeared to have the lowest gain and the largest variance. This is quantified in Fig. 1d.

Figure 1c quantifies the average endpoint variability as a function of target eccentricity and target size. Note that the bivariate contour ellipse area (BCEA), and thus endpoint scatter, increased with target size and with target eccentricity. We used a linear mixed model to confirm that these fixed effects were both statistically significant (*P* < 0.001, see Supplementary Table S1). There was also a significant interaction between target size and target eccentricity (*P* < 0.001). The differences in BCEA due to the different target sizes decrease at larger target eccentricities.

The mean and variance of the saccade gain were also affected by target size (Fig. 1d). We found that there was a statistically significant effect of target size and (reciprocal) target eccentricity, as well as a significant effect of the interaction between the two (see Supplementary Tables S2 and S3). Saccades made to the larger targets had a lower mean gain and higher gain variance. At larger target eccentricities, the mean gain was closer to 1 and the gain variance was closer to zero.

Note, that if we consider saccades of a certain amplitude, some of them were evoked by a target closer to the fovea whereas others were evoked by a target further away from the fovea (see Fig. 1a). As a result, some saccades of that particular amplitude were overshooting the target center (hypermetric saccades) while others of the same amplitude were undershooting the target center (hypometric saccades).

### Latency and gain

**Figure 2:**
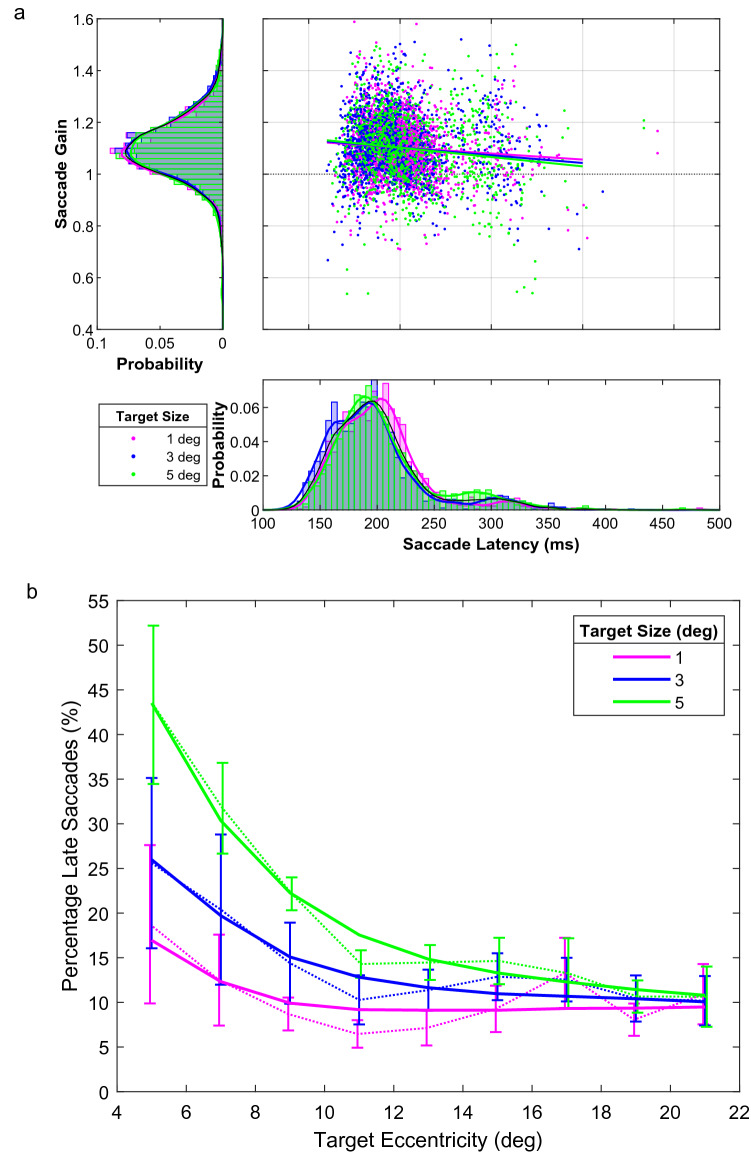
Latency and gain. (**a**) Scatter plot (center) showing saccade gain as a function of saccade latency for a single subject. Data are stratified by target size (color code). Solid lines are linear regression lines fitted to each of the data sets. The latency histograms (bottom) show bimodal distributions for a single subject with a group of early saccades and a group of late saccades. There are fewer late saccades than early ones. The gain histograms (left), which peak close to 1, illustrate the variability in saccade gain. This is also data from a single subject. (**b**) The percentage of saccades that were late for each target eccentricity and each target size. This is the average for all subjects. Error bars indicate ± 1 SEM.

Figure 2a shows latency and gain data from a single subject, pooled across target eccentricity. The latency histograms (bottom panel in Fig. 2a) reveal a bimodal distribution. For this participant, as well as most other participants (see Supplemental Fig. S1), we typically saw early saccades occurring with latencies less than ~ 250 ms and late responses occurring at latencies larger than that. It appears that the late saccades occurred more often when a 5° target was presented and early saccades occurred more often when saccades were made to a 1° target. Thus, target size affected the proportion of early and late responses. This can also be seen in Fig. 2b, which shows the proportion of early saccades averaged over all subjects. The curves in Fig. 2b were influenced, of course, by the 250 ms latency cut-off value that we adopted for this analysis, but we verified that the effects of target size and eccentricity on the percentage of late saccades held for a range of reaction time cutoffs.

The gain distributions (left panel in Fig. 2a) show that most of this subject’s saccades had a gain close to 1, meaning that the majority of saccades landed close to the center of the target. The scatter plot in the center panel of Fig. 2a shows the gain as a function of latency. We can see here that the gain decreases for responses with longer latencies. This trend is present for all three target sizes in this subject.

We used a mixed effects model to analyze the data from all subjects combined and found that saccade gain is significantly influenced by target size and target eccentricity (p < 0.05). The top panel of Fig. 1d illustrates these effects unadjusted for latency. The effect of latency on saccade gain varies significantly with target eccentricity (significant latency × eccentricity interaction; p < 0.05). The overall model had an r^2^ of 0.4 (Supplementary Table S4).

Late saccades occurred more often for the larger target sizes. This is shown in Fig. 2b, which plots the average percentage of late saccades occurring at each target eccentricity for each target size. There is a significant effect of target size (p < 0.005) on the percentage of late saccades and a significant interaction between the target size and (reciprocal) target eccentricity (p < 0.005). Target eccentricity on its own was not a significant factor, however (Supplementary Table S5).

### The main sequence

**Figure 3:**
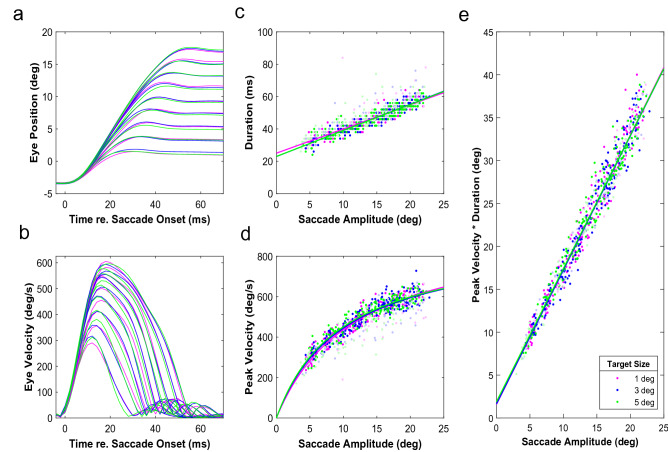
Main sequence. (**a**,**b**) Average eye position (**a**) and eye velocity (**b**) traces for saccades to each of the nine different target locations, stratified by target size (color code). (**c**,**d**) The main sequence relationships of duration (**c**) and peak velocity (**d**) against saccade amplitude for rightward saccades of the participant’s right eye. The faded dots represent the late saccades. Solid lines are fit to the data for saccades with latencies < 250 ms and a gain ranging between 0.9 and 1.1 (i.e., a subset of the brighter dots). (**e**) The Peak velocity*Duration is the same for early and late saccades.

Figure 3 shows the main-sequence data and the model fit lines for one of our participants. The duration data (Fig. 3c) was described with an affine function (Eq. 2) while the peak velocity (Fig. 3d) was described with a nonlinear function (Eq. 4). The model fit lines for the three different target sizes are close together in this participant. Note, however, that we only used normometric saccades with latencies < 250 ms when fitting the data (“Methods”) and that there are still significant deviations from the curves. The faded dots, which represent the late saccades, tend to deviate most from the main sequence fit. They are often slower and last longer. In line with previous work^31^, however, the product of peak velocity and duration of such slower saccades is still practically the same (Fig. 3e); there is a tight relationship between peak velocity times duration and saccade amplitude (Eq. 3).

In the remainder of this paper, we will continue to use the terms “early” and “late” saccades. It is important to emphasize, however, that all of the following regression analyses include latency as a continuous variable and are therefore *not* affected by the 250 ms reaction time criterion.

**Figure 4:**
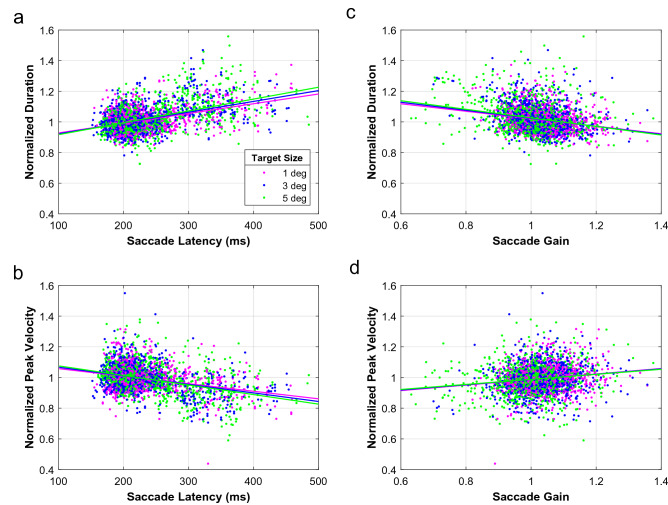
Normalized duration and peak velocity. Data in this plot are from the same individual (Subject Number 4) as in Fig. 3, and pooled across target location and recorded eye. The data are normalized with respect to the main sequence for normometric (i.e., 0.9 > gain > 1.1) saccades towards 1° wide targets with regular (< 250 ms) latencies (i.e., pink main sequence curves in Fig. 3). (**a**,**b**) Normalized duration and the normalized peak velocity as a function of the latency for each of the three target sizes (color coded). (**c**,**d**) Same kinematics data as in (**a**) and (**b**) but now plotted as a function of saccade gain.

To further explore how latency influenced the saccade kinematics and examine whether overshooting saccades had different kinematics from undershooting saccades of the same size (as they are evoked by targets at different locations), we first normalized saccade duration and peak velocity with respect to the main sequence of normometric saccades to 1° wide targets with typical latencies [“Methods”, Eqs. (5) and (6), respectively]. This normalization aims to eliminate the variation in saccade duration and peak velocity that is due to variations in saccade amplitude so that data can be pooled across saccades with different amplitudes. Figure 4 shows the normalized duration and peak velocity of saccades for a single participant (Subject Number 4) and how they are affected by saccade latency (Fig. 4a,b) and saccade gain (Fig. 4c,d). Note, that in this subject duration increases and peak velocity decreases when saccades have longer latencies. The opposite is true with gain. Durations decrease with increasing gain and peak velocities increase. As explained in the Introduction, such correlations with gain are expected if motor noise contributes to the variability in saccade endpoints. (Adjusted) R^2^ values are low (< 0.2 for the data in Fig. 4), but the observed patterns were consistent across subjects. The effect of latency on both the duration and peak velocity of saccades was statistically significant (p < 0.01) in all but two participants. The modulation with saccade gain was also statistically significant (p < 0.01) in all but two participants. Group statistics are listed in Supplementary Tables S6–S9.

**Figure 5:**
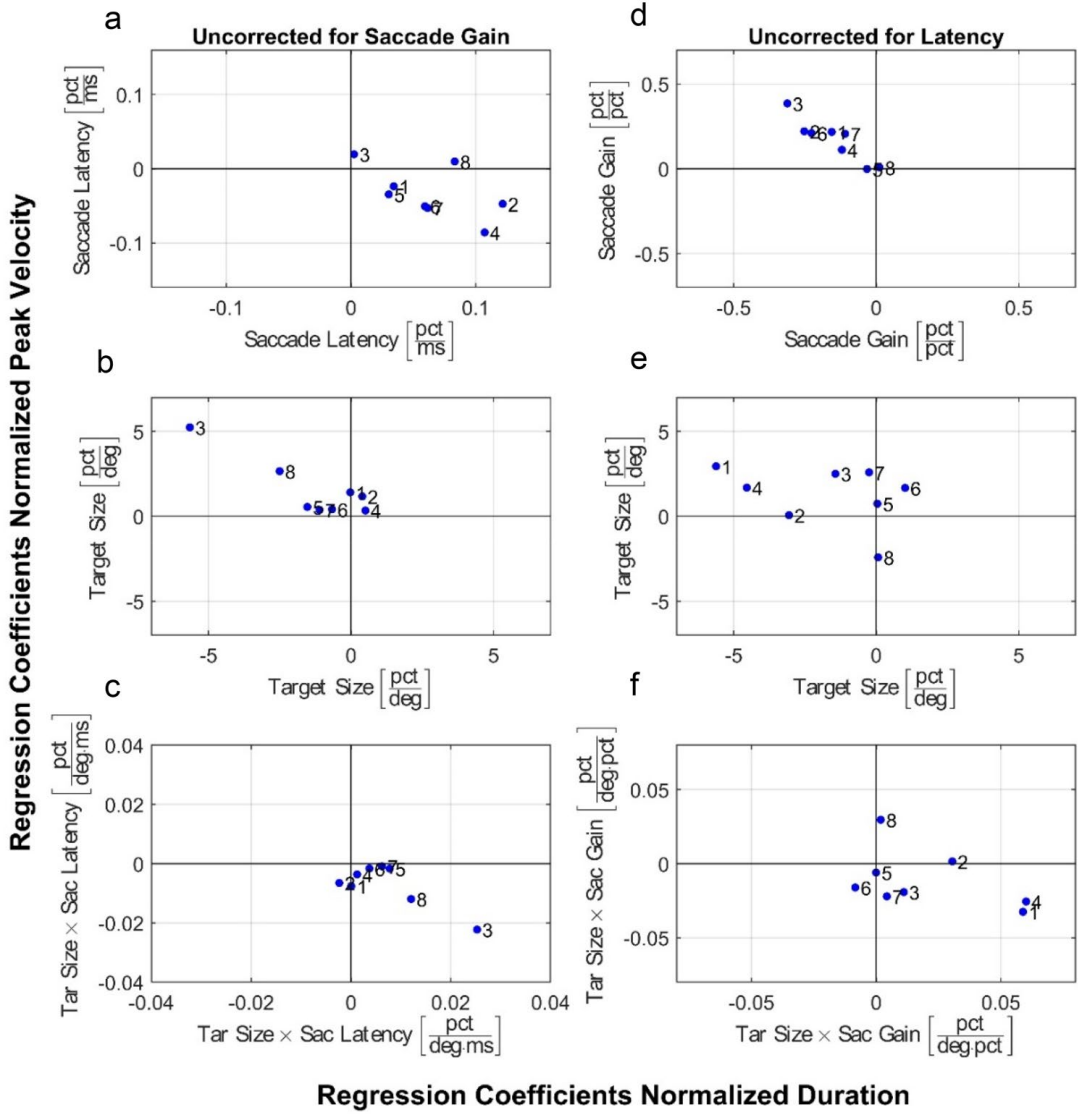
Normalized main sequence regression. Scatter plots of the regression coefficients which quantify how the normalized duration (horizontal axis) and the normalized peak velocity (vertical axis) depend either on target size and saccade latency (**a**–**c**) or on target size and saccade gain (**d**–**f**). Each numbered dot in the plots represents a single subject. (**a**–**c**) Regression coefficients when saccade latency, target size, and the interaction of the two are taken into account, and saccade gain is not. (as in Fig. 4a,b) The applied regression equations were (Wilkinson Notation): Normalized saccade duration ~ Target size + Saccade latency + Target size × Saccade latency. Normalized saccade peak velocity ~ Target size + Saccade latency + Target size × Saccade latency. (**d**–**f**) Regression results considering only saccade gain, target size, and the interaction of the two, but not saccade latency (as in Fig. 4c,d). The applied regression equations were: Normalized saccade duration ~ Target size + Saccade gain + Target size × Saccade gain, Normalized saccade peak velocity ~ Target size + Saccade gain + Target size × Saccade gain.

To illustrate that the influence of target size, saccade latency, saccade gain and their interactions on (normalized) saccade kinematics demonstrated a consistent pattern across subjects, Fig. 5 shows the coefficients of the regression lines seen in Fig. 4 for all subjects. Each numbered dot in the plots corresponds to one of our eight participants. The coefficients in the left column summarize the regression analysis illustrated in the left-hand panels of Fig. 4, which quantify the normalized kinematics as a function of target size and saccade latency without correcting for the influence of saccade gain. Likewise, the right column corresponds to the regression analysis in right-hand panels of Fig. 4, and shows how the normalized kinematics relate to target size and saccade gain without correcting for the effect of saccade latency. The top row plots show the slope of the line, the second row shows the intercept, and the third is the interaction term.

A qualitative assessment of these figures shows that there is a tendency for the coefficients to cluster in a certain quadrant. For example, in Fig. 5a most of the dots are in the bottom right quadrant. This tells us that the duration will increase with latency (positive slope) while the peak velocity will decrease with increasing latency (negative slope). Figure 5b shows that the coefficient for the target-size term is negative for duration and positive for peak velocity. This means that in the duration-latency plot (Fig. 4a) the intercept decreases with target size. For the peak velocity-latency plot (Fig. 4b), the intercept increases with target size. Figure 5c shows a positive interaction term for duration, and a negative interaction for peak velocity, which indicates that the effect of target size on the main sequence changes with latency. Late saccades to the largest target tend to have the longest duration and the lowest peak velocity, whereas the opposite is true for early saccades. The fact that most of the points cluster together indicates that the subjects’ main sequences all follow a similar pattern.

Linear mixed-effects regression analyses of the normalized kinematics data from all subjects indeed show that normalized saccade duration and normalized saccade velocity are significantly modulated by saccade latency and saccade gain and that these effects depend significantly on target size. The model including latency and target size accounted for 17% of the variation in normalized duration and for 15% of the variation in normalized peak velocity (Adjusted R^2^ values, Supplementary Tables S6 and S7). The model including saccade gain and target size accounted for 7% and 11% of the variance, respectively (Supplementary Tables S8 and S9). These are weak associations. In supplementary Figs. S2 and S3, we therefore include additional analyses of the normalized kinematics data to demonstrate that the reported effects were qualitatively consistent between individuals.

The analyses thus far have shown that duration and peak velocity are modulated by target size, saccade latency and their interactions as well as target size, saccade gain and their interactions even though the associations are weak. However, we have not yet considered further complexity.

**Figure 6:**
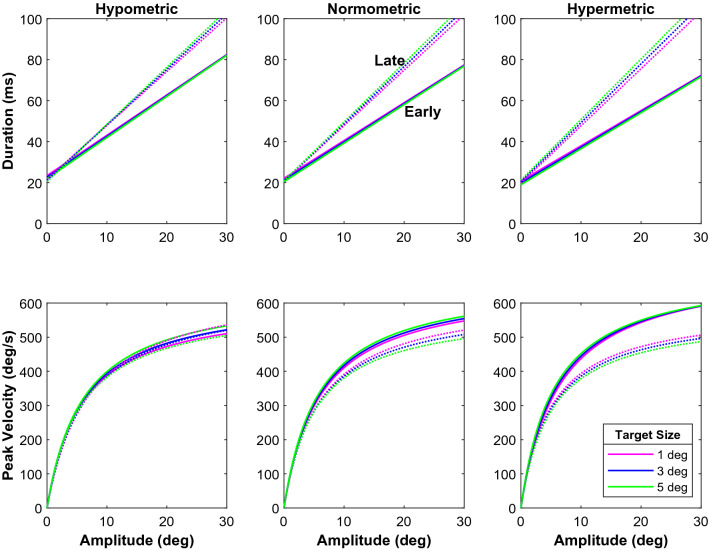
Effects of gain and latency on the main sequence. These plots show the relation between amplitude and duration and between amplitude and peak velocity that our respective regression models predict for different saccade gains, latencies and target sizes. Target size is color coded. The three columns are for different gains: hypometric (gain = 0.75), normometric (gain = 1.00), and hypermetric (gain = 1.25). The dotted lines represent the late saccades (latency 100 ms longer than the median latency) and the solid lines represent early saccades (latency 50 ms shorter than the median latency).

In our final analysis, we used a mixed-effects regression model to describe saccade duration and peak velocity as a function of saccade amplitude, saccade latency, and saccade gain, and tested if these relations are significantly modulated by target size. The resulting two models explained 85% of the variation in duration of the saccades and 89% of the variation in their peak velocity (Adjusted R^2^ values), respectively. Because they each had several significant two-way and three-way interaction terms (see Supplementary Tables S10 and S11), it is not easy to understand from the models’ coefficients how the main sequence relations are affected by saccade latency, saccade gain and target size. In Fig. 6, we therefore illustrate how gain and latency would impact the duration and velocity of saccades to the 1, 3 and 5° wide targets according to the fixed-effects terms in the model (marginal responses). Note that the late saccades (dotted lines) have longer durations and lower peak velocities. This difference is greater for the hypermetric saccades. For the hypometric and the normometric saccades we see a difference in durations between the early and late saccades, but this difference is not very pronounced for the peak velocity.

**Figure 7:**
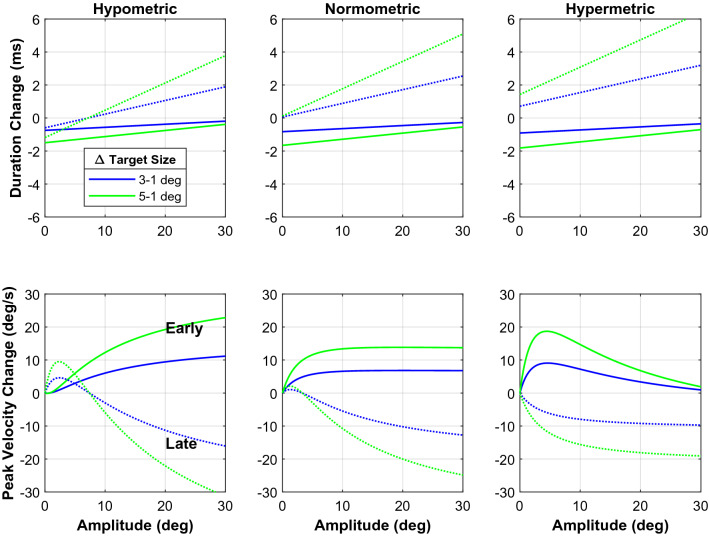
Main sequence differences between target sizes. Here we plot the change in duration and peak velocity between the target sizes. The blue lines show the difference between the 1° and the 3° target. The green lines show the difference between the 1° and the 5° target. Positive values indicate an increase in duration or peak velocity compared with saccades to the 1° wide targets.

In Fig. 7, we look into these differences further by plotting the change in duration and peak velocity between target sizes. The early saccades exhibit a positive duration change (the duration increases), while the opposite occurs for the late saccades. This pattern is consistent across the different kinds of gain. For the peak velocity, we see a positive logarithmic change for the early saccades. This change increases for the hypometric and normometric saccades. But for the hypermetric ones, we see an increase and then a decrease in change across amplitudes. The lines begin to converge around 30 deg. For the hypometric and normometric late saccades, we see a negative change in peak velocity that becomes larger with larger eccentricities. However, the hypometric late saccades initially show a positive increase and then begin to decrease around an amplitude of 3°.

## Discussion

Our data show that a simple manipulation of target size and target eccentricity in a single-target visual-evoked saccade task affects many aspects of saccade performance, including accuracy and precision, latency and the main-sequence properties. Our statistical analysis of these changes indicates that these effects are not independent, but interrelated. In what follows, we will discuss these different factors.

### Metrics

The observed effects of target size on saccade metrics (gain) were similar to those reported by Ploner et al. ^23^. We saw more saccade endpoint scatter for larger targets (higher BCEA) and the gain was lower and more variable for the largest target than for smaller targets (see Fig. 1c,d). Larger targets also had a larger total luminance as a consequence of keeping the peak luminance fixed (“Methods”). We cannot exclude that this affected the way in which target size influenced the endpoint variability. However, we would not expect the effects to differ fundamentally had we changed the peak luminance with target size to keep the total luminance constant. In any case, as intended with our stimulus manipulation, saccades became less precise and less accurate when made to larger targets. However, these effects were mainly seen at target eccentricities less than 12°. At larger target eccentricities, the differences in saccade accuracy and precision between target sizes diminished. It is possible that the decreased retinal resolution at larger eccentricities^32,33^ causes saccades into the peripheral visual field to exhibit similar levels of accuracy and precision, regardless of target size. Thus, if the level of uncertainty about target borders for all three target sizes is relatively equal in the retinal periphery, saccades would be equally inaccurate.

### Latency

We noticed an effect of target size on latency, where larger targets elicited more late saccades, but only at small target eccentricities (Fig. 2b). A previous study by De Vries et al.^34^ also reported that saccade latency depends on target size, referred to as the size-latency phenomenon. They observed longer latencies when saccades were made to larger targets and that this effect was strongest when the target overlapped with the central visual field. Our results are in line with these findings.

At large target eccentricities, we no longer observed an effect of target size on latency (Fig. 2b). Ploner et al.^23^ suggested that this may be related to the role of fixation cells in the superior colliculus. The area of the SC that programs small saccades overlaps with these fixation cells. Thus, a larger population of fixation cells would be activated for large targets at small eccentricities, slowing down saccade initiation. Such an effect would disappear at larger eccentricities. This may explain why we only observed longer latencies for the larger targets at small eccentricities.

We found that the latency difference between early saccades and late saccades was about 100 ms. Note, that this was also the duration of the visual target stimuli. Thus, an alternative explanation for the late saccades could be that they were triggered by the *offset* of the target. Bimodal latency distributions have been associated with fine operant control of saccadic latency, supporting the hypothesis of a cost–benefit optimization of saccade latencies^35^. Interestingly, our findings show that the variability in saccade kinematics is linked to the variability in saccade latency.

### Main sequence

Our results show that saccade velocity and saccade duration do not depend on saccade amplitude alone, but are modulated by several factors. We found that target size, saccade latency, saccade gain, and interactions between these factors all affected the main-sequence behavior (see Figs. 6 and 7, and Supplementary Tables S10 and S11). Despite this complexity, a clear picture emerged. Early saccades had shorter durations and higher peak velocities than late saccades of the same amplitude to the same target. Peak velocities and durations of early saccades also varied strongly with saccade gain. Early hypermetric saccades were faster and had shorter durations than hypometric responses of the same amplitude. In addition, the kinematics of early saccades followed the target-size dependency predicted for an optimal speed-accuracy trade-off. Early saccades of a given amplitude had shorter durations and higher peak velocities for larger targets than for smaller targets. In line with the accuracy data, this effect of target size varied with saccade amplitude and saccade gain. Interestingly, for late saccades we found the opposite pattern: Late saccades to a large target had longer durations and lower peak velocities than late saccades to a small target at the same eccentricity. Note, that this pattern emerged from the data without explicitly modeling a dissociation between early and late saccades; in all our analyses of the saccade kinematics (Figs. 4, 5, 6, 7), we treated latency as a continuous variable.

Although the effects of target size on the kinematics of saccades were modest compared to the effects on saccade latency and saccade gain, the observed modulation in peak velocity of about 10°/s and about 2–4 ms in duration were comparable to those reported in previous studies. For instance, Xu-Wilson et al.^8^ found a 5°/s increase in peak velocity and 1–2 ms decrease in duration when subjects made saccades to images of faces. Similarly, Reppert et al.^7^ observed an approximate increase of 15°/s when subjects made saccades to faces instead of dots. In the 2012 study by Muhammed et al.^36^ subjects were told to make fast or slow saccades to targets on different trials. They saw an increase in peak velocity of about 8°/s for fast saccades, and a similar decrease for slow saccades, relative to direct visual-evoked control responses. In Montagnini and Chelazzi’s^37^ study, subjects made saccades to a target while completing a letter-discrimination task under time constraints. They found that this “perceptual urgency” task caused an average increase in peak velocity of about 35°/s compared to a control task^37^.

### Multiple pathways?

It is not immediately clear why the effect of target size was opposite for early versus late saccades. One could speculate that the locations of small targets were harder to remember than those of large targets, which activate more neural tissue. If true, the uncertainty about target location could indeed become reversed over time and therefore late saccades could be planned more precisely to large targets than to small targets. Future experiments might test this idea by keeping the target visible until the saccade starts. This would still ensure that saccades are executed open-loop (i.e., no visual feedback), but the late saccades cannot be classified as potentially memory-guided. Memory-guided saccades are known to be slower than visually-guided saccades^5^. Thus, one could interpret the observed effects of saccade latency on kinematics as a transition from a visually guided to a memory-guided saccade.

Our data revealed two distributions of saccade latencies: early saccades, below about 250 ms and late saccades, above 250 ms (Fig. 2a, bottom panel; Supplemental Materials, Fig. S1). As these two saccade populations were differentially affected by the stimulus manipulations, it could hint at the possibility that two different subsystems govern the early versus the late saccades. Lesion studies in monkey have indicated that saccade signals may indeed be routed over two parallel pathways: a path via the superior colliculus (SC) to the brainstem, and a cortical pathway from the frontal eye fields (FEF) to the brainstem that bypasses the SC^38^. Recently, reversible FEF cooling indicated that visual-evoked, fast saccades follow the collicular route, while the latter pathway could be involved in late (memory-guided) saccade responses^39^.

Previous behavioral studies have postulated that distinct saccade latency distributions reflect the different stages of neural processes underlying saccade planning and execution. Fischer et al.^40^ proposed that so-called express saccades (extremely short latencies of about 100–135 ms) occur after the disengagement of attention from the fixation target, early saccades (140–180 ms) after the decision to make an eye movement, and late saccades (> 200 ms) after the computation of saccade metrics. In a study on the susceptibility of saccades to visual illusions, de’Sperati and Baud-Bovy^41^ surmised that short-latency saccades (~ 150 ms) occur after fast cortical visual processing, while longer latency saccades (~ 250) wait for slow cortical feedback loops to shape the sensory input.

### Variability

In their theoretical work, Harris and Wolpert^3,10^ assumed that the saccadic system minimizes the duration of saccades while accounting for the consequences of signal-dependent noise in its control signals. Signal-dependent noise has been observed in the firing patterns of motor units^42,43^ and in saccade-related neurons of the superior colliculus^4^. Van Beers^13^ pointed out, however, that additive noise might play an important role too since it would reduce the accuracy of slow saccades that last longer. His analysis indicated that the optimal saccade duration may be understood from a combination of signal-dependent noise favoring long durations, and constant noise, which prefers short durations.

Note, however, that the time needed to reach the target is determined by the sum of saccade latency and saccade duration. In fact, saccade latencies are typically long and more variable compared to the saccade durations and therefore will have a bigger impact on how soon a target is reached than saccade duration. Perhaps this is one of the reasons why saccade latency had such a strong effect on saccade kinematics in our study. After all, minimizing the duration of a saccade could become less of a priority for the saccadic system if it has already decided to make the movement late. Put another way: why bother making a saccade as fast and accurately as possible if you are going to be late anyway? This broader perspective on response optimization could explain, at least partly, why late saccades were typically slower and lasted longer than early saccades, and therefore failed to follow the predictions of the Harris and Wolpert speed-accuracy trade-off model that considers only the saccade duration.

Smeets and Hooge^25^ also studied the variability in saccade kinematics, albeit without stimulus manipulations. Starting from a simple pulse-step control model, and assuming that saccade duration depends only on the duration (and not the amplitude) of the motor command, they reasoned that variations in pulse height due to motor noise (pulse-height noise) would lead to systematic deviations from the main sequence. A higher than normal pulse would result in saccadic overshoot with a higher than normal peak velocity, whereas a lower than normal pulse would result in an undershoot with a lower than normal peak velocity. In this simplified open-loop scheme, pulse duration and consequently movement duration would be set deterministically by the amplitude of the intended movement (and not by the actual movement). The intended movement, however, is an internal saccade plan that cannot be measured directly and need not be identical for saccades to the same target in different trials. It may also be affected by noise in target localisation, e.g., due to misperception of the initial eye position, noise in the visual system, and noise in the collicular motor map^4,24^. Yet, localisation noise by itself would keep saccades on the main sequence. Our datasets contained amplitude-matched saccades that overshot one target and undershot another (more eccentric) target. According to Smeets and Hooge, if the variability in overshoots and undershoots resulted exclusively from localisation noise, saccades with different gains should have followed the same main sequence relation (after accounting for the effects of saccade latency and target size)^25^. We found, however, that both peak velocity and duration of amplitude-matched saccades were modulated by saccade gain: saccades with higher gains had higher peak velocities and shorter durations while saccades with lower gains had lower peak velocities and longer durations. Such correlations with saccade gain (that do not result from variations in saccade amplitude) are in line with the pulse-height noise hypothesis. This suggests that the differences in saccade kinematics that we saw for overshooting and undershooting saccades were at least partly explained by motor noise.

## Conclusion

Our findings are consistent with the premise that the variability in saccade endpoints (which increased with target size) does not result from localisation noise alone, but that motor noise has a significant influence on the saccade trajectories as well. In addition to the variability in saccade kinematics that may be explained by motor noise, we found that the main-sequence relations were also modulated by saccade latency and target size, both of which may impact the accuracy constraints for executing a given saccade. The effects of target size on the kinematics of early saccades supports the theory that the saccadic system weighs the detrimental effects of motor noise on saccade accuracy against movement duration and speed, but this is not the case for late saccades. The apparent discrepancy between early and late saccades may relate to different saccadic subsystems being involved.

## Supplementary Information

Supplementary Information.
