# Supplementary material for: Speed-accuracy tradeoffs influence the main sequence of saccadic eye movements
Source: Sci Rep. 2022 Mar 28;12:5262. doi: 10.1038/s41598-022-09029-8 (PMC8960849; doi:10.1038/s41598-022-09029-8)
Supplement: Supplementary file 1 — Supplementary Information. [file 41598_2022_9029_MOESM1_ESM.docx]

**Speed-accuracy tradeoffs influence the main sequence of saccadic eye movements**

Leslie Guadron^1^, John van Opstal^2^, Jeroen Goossens^1*^

^1^ Department of Cognitive Neuroscience, Donders Institute for Brain, Cognition and Behaviour, Radboudumc, P.O. Box 9101, 6500 HB, Nijmegen, The Netherlands

^2^ Department of Biophysics, Donders Institute for Brain, Cognition and Behaviour, Radboud University, P.O. Box 9010//066, 6500 GL, Nijmegen, The Netherlands

^*^ Corresponding author: [J.Goossens@donders.ru.nl](mailto:J.Goossens@donders.ru.nl)

SUPPLEMENTARY MATERIALS


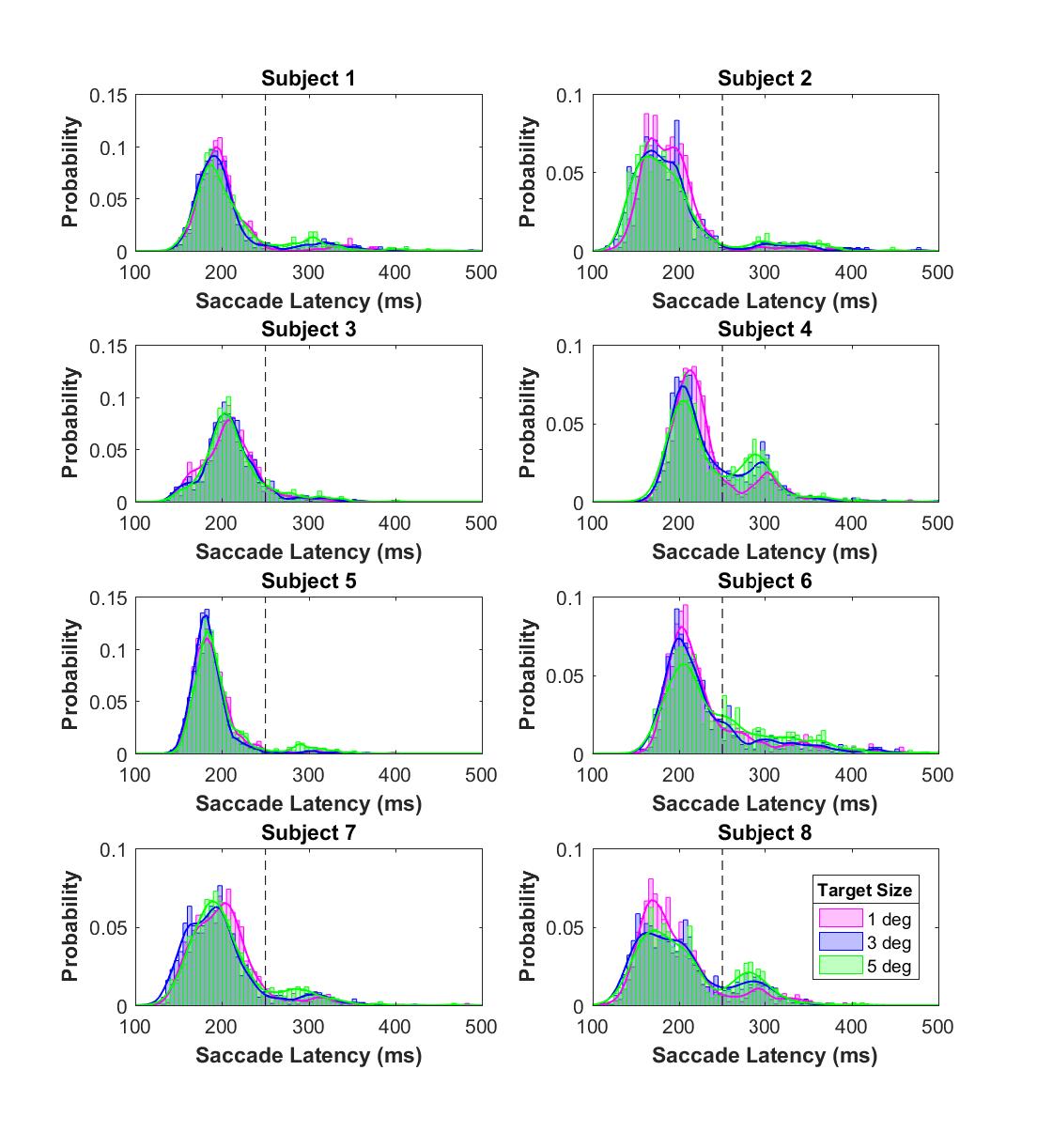


**Figure S1: Individual Latency Histograms**

These are the latency distributions for each individual subject. The different colors are for the three target sizes we presented. Data are pooled data across saccade amplitude and direction (binwidth 5 ms). Note scaling differences between ordinates. Most subjects show a bimodal distribution with a separate population of early and late saccades. The black dashed line indicates the fixed 250 ms latency cut off value that we have used to quantify the percentage of late saccades in Figure 2B and to dissociate between early and late saccades in Figure 3.


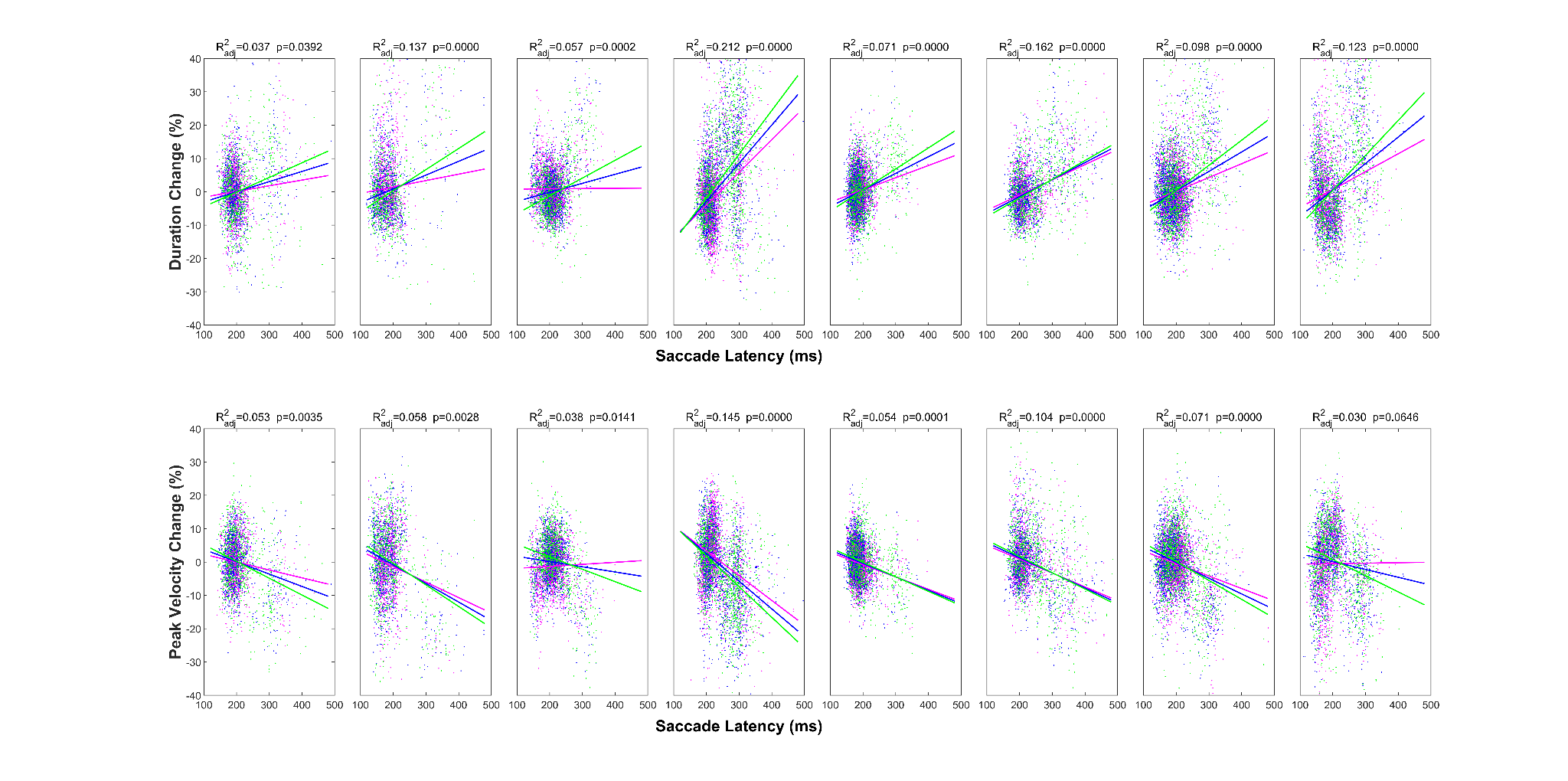


**Figure S2: Effect of latency on normalized duration and peak velocity in individual subjects**

Percent change in duration (top) and peak velocity (bottom) as a function of latency for all eight participants. Data were normalized with respect to the participant’s main sequence of normometric saccades to 1 degree wide targets with typical latencies (see Methods) and then adjusted for the effect of saccade gain (pooled across target size). Linear regression lines fitted to the data (pooled across eyes and target location) show that durations tend to increase with latency (positive slopes) while peak velocities tend to decrease with latency (negative slopes) in all subjects. We also see that the slopes and intercepts of the regression lines tend to be different for the three target sizes (pink: 1 deg; blue: 3 deg; green: 5 deg) in such a way that durations decrease and peak velocities increase with target size for early saccades, whereas the opposite is true for late saccades, except in the third participant (3rd column). All fits were obtained with Matlab’s fitlm function using its default 'bisquare' weight function.


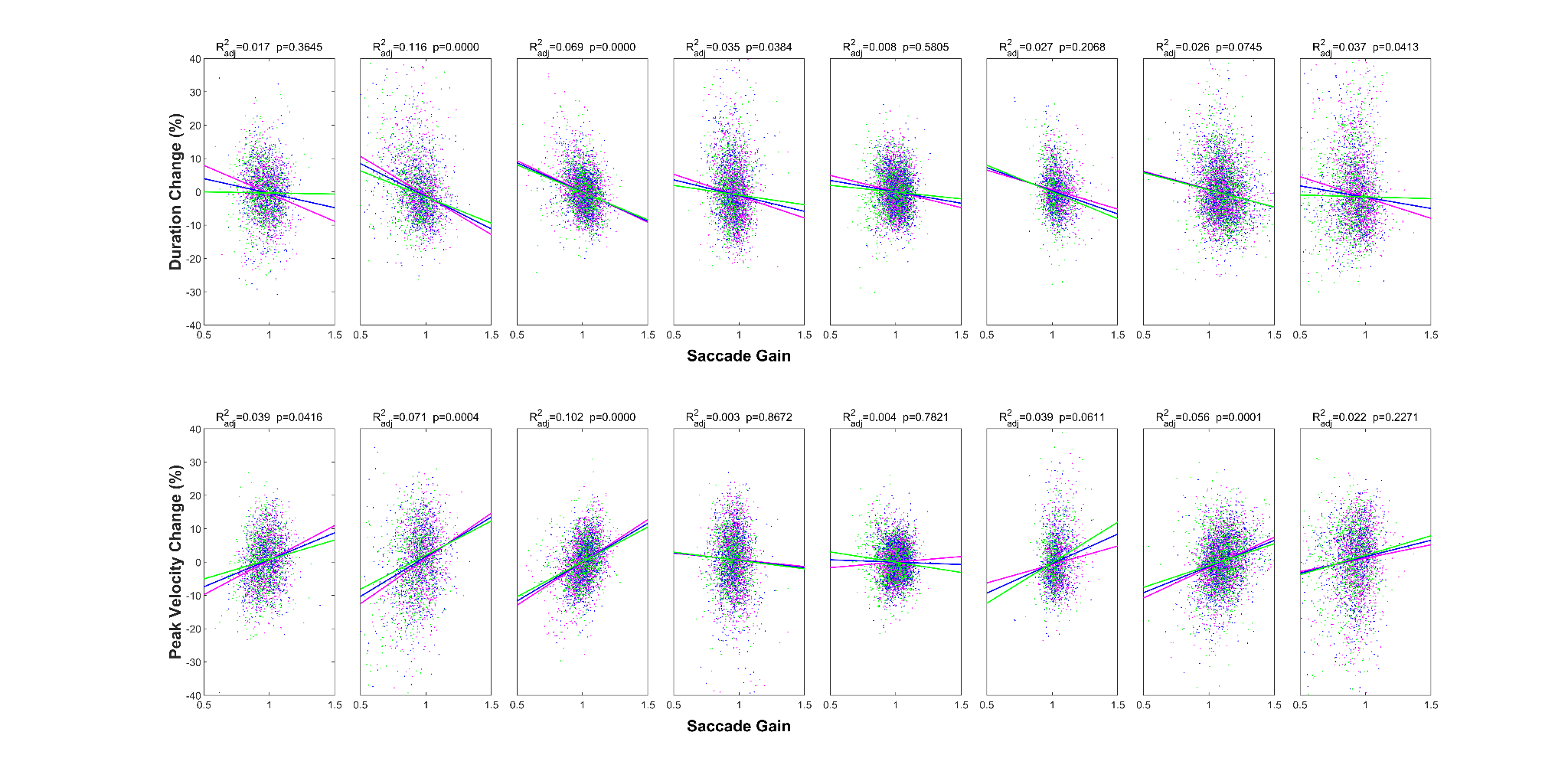


**Figure S3: Effect of saccade gain on normalized duration and peak velocity in individual subjects**

Percent changes in duration (top) and peak velocity (bottom) as a function of saccade gain for “early” saccades (latency < 250 ms) in all eight participants. Data were normalized with respect to the participant’s main sequence of normometric saccades to 1 degree wide targets with typical latencies (see Methods) and then adjusted for the target-size dependent effects of latency. Linear regression lines fitted to the data (pooled across eyes and target location) show that duration tends to decrease with gain (negative slopes) while peak velocity tends to increase with gain (positive slopes, except in subjects 4 and 5 whose fits were not significant). Both effects tend to increase with target size (pink: 1 deg; blue: 3 deg; green: 5 deg) as indicated by the steeper slopes. Crossing points of the regression lines are difficult to interpret in these plots because average gains also vary with target size and eccentricity (see Figure 1). All fits were obtained with Matlab’s fitlm function using its default 'bisquare' weight function.

Names and definition of variables used in the statistical analyses shown in Supplementary Tables S1-S11

| Continuous model variable | Definition |
| --- | --- |
| TGSize | target size |
| ECC | target eccentricity |
| invECC | 1/target eccentricity |
| Latency | reaction time relative to target onset |
| Gain | saccade amplitude/retinal target eccentricity |
| Amplitude | saccade amplitude |
| invAmplitude | 1/saccade amplitude |

| Grouping variable | Definition |
| --- | --- |
| Subj | subject identifier |
| DIR | saccade direction (left or right) |
| Eye | recorded eye (left or right) |

**Table S1: Bivariate Contour Ellipse Area (Figure 1C)**

Linear mixed-effects regression (Matlab function fitlme) showing statistically significant fixed-effects of target eccentricity and target size on the BCEA. The regression included BCEA values from n=864 observations: 3 target sizes * 9 eccentricities * 2 directions * 8 subjects * 2 eyes.

Fixed effects coefficients (95% CIs):

Name Estimate SE tStat DF pValue Lower Upper

{'(Intercept)’} 0.077541 0.090354 0.85818 860 0.39103 -0.0998 0.25488

{'TGSize' } 0.12802 0.022178 5.7724 860 1.0911e-08 0.084491 0.17155

{'ECC' } 0.087351 0.0072962 11.972 860 1.1679e-30 0.073031 0.10167

{'TGSize:ECC' } -0.0063814 0.0016907 -3.7743 860 0.00017147 -0.0096998 -0.0030629

Number of observations = 864

Rsquare Adjusted = 0.7728

**Table S2: Gain Mean (Figure 1D)**

Same analysis as in Table S1 but now showing statistically significant fixed-effects of target eccentricity and target size on the Gain mean. The declining effect of target eccentricity was adequately captured by assuming an inverse linear relationship with target eccentricity.

Fixed effects coefficients (95% CIs):

Name Estimate SE tStat DF pValue Lower Upper

{'(Intercept)' } 0.97792 0.011359 86.093 860 0 0.95563 1.0002

{'TGSize' } 0.0059482 0.001266 4.6985 860 3.0496e-06 0.0034634 0.0084329

{'invECC' } 0.28048 0.10179 2.7556 860 0.0059826 0.080702 0.48026

{'TGSize:invECC'} -0.13303 0.015163 -8.7732 860 9.2109e-18 -0.16279 -0.10327

Number of observations = 864
Rsquared Adjusted = 0.9137

**Table S3: Gain Variance (Figure 1D)**

Same analysis as in Table S1 but now showing statistically significant fixed-effects of target eccentricity and target size on the Gain variance.

Fixed effects coefficients (95% CIs):

Name Estimate SE tStat DF pValue Lower Upper

{'(Intercept)' } 0.0022125 0.0010266 2.1552 860 0.031422 0.00019761 0.0042275

{'TGSize' } -0.0011492 0.00024429 -4.7041 860 2.9691e-06 -0.0016287 -0.00066971

{'invECC' } 0.050392 0.011053 4.5593 860 5.8762e-06 0.028699 0.072085

{'TGSize:invECC'} 0.021669 0.0030145 7.1884 860 1.4236e-12 0.015753 0.027586

Number of observations = 864
Rsquared Adjusted = 0.7799

**Table S4: Gain and Latency (Figure 2A)**

Linear mixed-effects regression (fitlme) quantifying saccade gain as a function of saccade latency, target eccentricity and target size. The regression included Gain values from n=32704 observations: 3 target sizes * 9 eccentricities * 2 directions * 8 subjects * 2 eyes * typically 30+ trial repetitions.

Fixed effects coefficients (95% CIs):

Name Estimate SE tStat DF pValue Lower Upper {'(Intercept)' } 1.0442 0.015992 65.291 32696 0 1.0128 1.0755

{'Latency' } -0.00028763 0.0001981 -1.452 32696 0.14652 -0.00067591 0.00010065

{'TGSize' } 0.0028866 0.0010271 2.8105 32696 0.0049496 0.00087347 0.0048996

{'ECC' } -0.0031216 0.00054893 -5.6867 32696 1.3059e-08 -0.0041976 -0.0020457

{'invECC' } 0.03006 0.07815 0.38465 32696 0.7005 -0.12312 0.18324

{'Latency:ECC' } 2.2328e-05 9.7781e-06 2.2835 32696 0.022407 3.163e-06 4.1494e-05

{'TGSize:invECC' } -0.080613 0.013605 -5.9251 32696 3.1519e-09 -0.10728 -0.053946

{'Latency:TGSize:invECC'} -0.00059239 0.00013534 -4.3771 32696 1.2062e-05 -0.00085766 -0.00032713

Number of observations = 32353
Rsquared Adjusted = 0.4124

**Table S5: Percent Late Saccades (Figure 2B)**

General linear mixed-effects regression quantifying the number of late saccades (fitglme with a binomial distribution) as a function of target eccentricity and target size.

Fixed effects coefficients (95% CIs):

Name Estimate SE tStat DF pValue Lower Upper

{'(Intercept)'} -2.3408 0.29472 -7.9425 860 6.1867e-15 -2.9193 -1.7624

{'TGSize' } -0.15317 0.045686 -3.3528 860 0.00083497 -0.24284 -0.063506

{'invECC' } -4.3798 3.2736 -1.3379 860 0.18128 -10.805 2.0454

{'TGSize:invECC' } 3.8168 0.43492 8.776 860 9.0052e-18 2.9632 4.6705

Number of observations = 32704
Rsquared Adjusted = 0.9390

**Table S6: Normalized Duration and Latency (Figure 4A)**

Linear mixed-effects regression analysis (fitlme) quantifying normalized saccade duration as a function of saccade latency and target size. The regression included data from n=32353 observations: 3 target sizes * 9 eccentricities * 2 directions * 8 subjects * 2 eyes * typically 30+ trial repetitions. Saccades with atypical, double-peaked velocity profiles were excluded.

Fixed effects coefficients (95% CIs):

Name Estimate SE tStat DF pValue Lower Upper

{'(Intercept)' } 1.0054 0.0017093 588.21 32349 0 1.0021 1.0088

{'Latency' } 0.00063967 7.8359e-05 8.1633 32349 3.3766e-16 0.00048608 0.00079325

{'TGSize' } -0.00017583 0.00085789 -0.20495 32349 0.83761 -0.0018573 0.0015057

{'Latency:TGSize'} 5.5209e-05 1.2866e-05 4.2909 32349 1.7845e-05 2.999e-05 8.0427e-05

Number of observations = 32353

Rsquared Adjusted = 0.1719

**Table S7: Normalized Peak Velocity and Latency (Figure 4B)**

Same analysis as in Table S6 but now on normalized peak velocities.

Fixed effects coefficients (95% CIs):

Name Estimate SE tStat DF pValue Lower Upper

{'(Intercept)' } 0.99716 0.0016304 611.62 32349 0 0.99396 1.0004

{'Latency' } -0.00033954 7.8009e-05 -4.3525 32349 1.3498e-05 -0.00049244 -0.00018664

{'TGSize' } 0.0016382 0.00076748 2.1345 32349 0.032807 0.00013392 0.0031425

{'Latency:TGSize'} -5.5518e-05 1.0773e-05 -5.1536 32349 2.5704e-07 -7.6633e-05 -3.4403e-05

Number of observations = 32353

Rsquared Adjusted = 0.1552

**Table S8: Normalized Duration and Gain (Figure 4C)**

Linear mixed-effects regression analysis (fitlme) quantifying normalized saccade duration as a function of saccade gain and target size. The regression included data from n=32353 observations: 3 target sizes * 9 eccentricities * 2 directions * 8 subjects * 2 eyes * typically 30+ trial repetitions. Saccades with atypical, double-peaked velocity profiles were excluded.

Fixed effects coefficients (95% CIs):

Name Estimate SE tStat DF pValue Lower Upper

{'(Intercept)'} 1.1675 0.012876 90.672 32349 0 1.1422 1.1927

{'Gain' } -0.15375 0.012915 -11.905 32349 1.3055e-32 -0.17906 -0.12843

{'TGSize' } -0.017304 0.0067251 -2.573 32349 0.010086 -0.030485 -0.0041223

{'Gain:TGSize'} 0.019942 0.0071295 2.7972 32349 0.0051585 0.0059683 0.033916

Number of observations = 32353

Rsquared Adjusted = 0.0764

**Table S9: Normalized Peak Velocity and Gain (Figure 4D)**

Same analysis as in Table S8 but now on normalized peak velocities.

Fixed effects coefficients (95% CIs):

Name Estimate SE tStat DF pValue Lower Upper

{'(Intercept)'} 0.79477 0.021904 36.283 32349 1.3875e-282 0.75184 0.8377

{'Gain' } 0.19745 0.022627 8.7265 32349 2.7504e-18 0.1531 0.2418

{'TGSize' } 0.013532 0.0028993 4.6673 32349 3.0638e-06 0.0078493 0.019215

{'Gain:TGSize'} -0.01301 0.0030187 -4.3097 32349 1.6397e-05 -0.018926 -0.0070928

Number of observations = 32353

Rsquared Adjusted = 0.1064

**Table S10: Regression model for saccade duration (Figure 6, top)**

Linear mixed-effects regression analysis (fitlme) quantifying saccade duration as a function of saccade amplitude, saccade gain, saccade latency and target size with interactions. The regression included data from n=32353 observations: 3 target sizes * 9 eccentricities * 2 directions * 8 subjects * 2 eyes * typically 30+ trial repetitions. Saccades with atypical velocity profiles were excluded. Latencies were centered on the participants’ median latency. Interaction terms that were not statistically significant were dropped from the analysis. Random effects are reported to indicate the variability between subjects conditioned on saccade direction and eye.

Fixed effects coefficients (95% CIs):

Name Estimate SE tStat DF pValue Lower Upper

{'(Intercept)' } 27.023 0.9434 28.644 32340 3.1817e-178 25.174 28.872

{'Latency' } -0.011932 0.0049487 -2.4111 32340 0.015908 -0.021632 -0.0022324

{'Amplitude' } 2.3347 0.12293 18.991 32340 5.486e-80 2.0937 2.5756

{'Gain' } -5.1258 0.90884 -5.6399 32340 1.7156e-08 -6.9072 -3.3444

{'TGSize' } -0.59484 0.17551 -3.3893 32340 0.00070162 -0.93883 -0.25084

{'Amplitude:Gain' } -0.22298 0.094725 -2.354 32340 0.018577 -0.40865 -0.03732

{'Latency:TGSize' } -0.0068095 0.0016403 -4.1514 32340 3.3125e-05 -0.010025 -0.0035945

{'Amplitude:TGSize' } 0.019993 0.0038237 5.2288 32340 1.7167e-07 0.012499 0.027488

{'Gain:TGSize' } 0.32853 0.17505 1.8768 32340 0.060559 -0.014576 0.67163

{'Latency:Amplitude:Gain' } 0.0055337 0.0015756 3.5121 32340 0.00044516 0.0024455 0.008622

{'Latency:Amplitude:TGSize'} 0.00021412 9.3078e-05 2.3004 32340 0.021431 3.1681e-05 0.00039655

{'Latency:Gain:TGSize' } 0.0097754 0.0017026 5.7416 32340 9.4622e-09 0.0064383 0.013112

Random effects covariance parameters (95% CIs):

Group: Subj:DIR:Eye (32 Levels)

Name1 Name2 Type Estimate Lower Upper

{'(Intercept)' } {'(Intercept)' } {'std'} 2.2167 1.4331 3.4285

{'Latency' } {'Latency' } {'std'} 0.019697 0.014519 0.026723

{'Amplitude' } {'Amplitude' } {'std'} 0.585 0.44308 0.77236

{'Gain' } {'Gain' } {'std'} 1.8408 1.0518 3.2216

{'TGSize' } {'TGSize' } {'std'} 0.14697 0.103 0.20973

{'Latency:Amplitude' } {'Latency:Amplitude' } {'std'} 0.0079969 0.0058638 0.010906

{'Amplitude:Gain' } {'Amplitude:Gain' } {'std'} 0.38893 0.28317 0.53419

{'Latency:Amplitude:Gain' } {'Latency:Amplitude:Gain' } {'std'} 0.0080044 0.005928 0.010808

{'Latency:Amplitude:TGSize'} {'Latency:Amplitude:TGSize'} {'std'} 0.00027493 0.00019083 0.00039608

Number of observations = 32353
Rsquared Adjusted = 0.8515

**Table S11: Regression model for peak velocity (Figure 6, bottom)**

General linear mixed-effects regression analysis (fitglme with a reciprocal link function) quantifying peak velocity as a function of saccade amplitude, saccade gain, saccade latency and target size. The regression included data from n=32353 observations: 3 target sizes * 9 eccentricities * 2 directions * 8 subjects * 2 eyes * typically 30+ trial repetitions. Saccades with atypical velocity profiles were excluded. Latencies were centered on the participants’ median latency. Interaction terms that were not statistically significant were dropped from the analysis. Random effects are reported to indicate the variability between subjects conditioned on saccade direction and eye.

Fixed effects coefficients (95% CIs):

Name Estimate SE tStat DF pValue Lower Upper

{'(Intercept)' } 0.0017803 9.991e-05 17.819 32337 1.0892e-70 0.0015845 0.0019762

{'Latency' } -6.1583e-06 9.6404e-07 -6.388 32337 1.704e-10 -8.0478e-06 -4.2687e-06

{'invAmplitude' } 0.011333 0.00046546 24.348 32337 8.9338e-130 0.010421 0.012246

{'Gain' } -0.00023651 6.9325e-05 -3.4115 32337 0.00064677 -0.00037239 -0.00010063

{'TGSize' } -1.9293e-05 8.0253e-06 -2.404 32337 0.01622 -3.5023e-05 -3.5633e-06

{'Latency:invAmplitude'} 3.3987e-05 8.0477e-06 4.2231 32337 2.416e-05 1.8213e-05 4.976e-05

{'Latency:Gain' } 6.4731e-06 9.5857e-07 6.7529 32337 1.4732e-11 4.5943e-06 8.352e-06

{'invAmplitude:Gain' } -0.0019365 0.00043373 -4.4648 32337 8.042e-06 -0.0027867 -0.0010864

{'Latency:TGSize' } 9.0988e-07 2.5019e-07 3.6368 32337 0.00027651 4.195e-07 1.4003e-06

{'Gain:TGSize' } 2.3554e-05 7.9732e-06 2.9542 32337 0.0031369 7.9267e-06 3.9182e-05

{'Latency:invAmplitude:Gain' } -3.1587e-05 8.468e-06 -3.7302 32337 0.00019169 -4.8185e-05 -1.4989e-05

{'Latency:invAmplitude:TGSize'} -8.6047e-06 1.8533e-06 -4.6429 32337 3.4491e-06 -1.2237e-05 -4.9721e-06

{'Latency:Gain:TGSize' } -6.8191e-07 2.4793e-07 -2.7504 32337 0.0059553 -1.1679e-06 -1.9596e-07

{'invAmplitude:Gain:TGSize'} -0.00011054 2.4715e-05 -4.4726 32337 7.7536e-06 -0.00015899 -6.2099e-05

{'Latency:invAmplitude:Gain:TGSize'}8.8147e-06 1.9994e-06 4.4087 32337 1.0432e-05 4.8958e-06 1.2734e-05

Random effects covariance parameters:

Group: Subj:DIR:Eye (32 Levels)

Name1 Name2 Type Estimate

{'(Intercept)' } {'(Intercept)' } {'std'} 0.00051202

{'Latency' } {'Latency' } {'std'} 5.6939e-07

{'invAmplitude' } {'invAmplitude' } {'std'} 0.0017603

{'Gain' } {'Gain' } {'std'} 0.00031074

{'TGSize' } {'TGSize' } {'std'} 6.9492e-06

{'Latency:invAmplitude' } {'Latency:invAmplitude' } {'std'} 3.2036e-06

{'Latency:Gain' } {'Latency:Gain' } {'std'} 4.7618e-07

{'invAmplitude:Gain' } {'invAmplitude:Gain' } {'std'} 0.0014627

{'Latency:TGSize' } {'Latency:TGSize' } {'std'} 1.8719e-07

{'Gain:TGSize' } {'Gain:TGSize' } {'std'} 6.9481e-09

{'Latency:invAmplitude:Gain' } {'Latency:invAmplitude:Gain' } {'std'} 5.7703e-06

{'Latency:invAmplitude:TGSize' } {'Latency:invAmplitude:TGSize' } {'std'} 5.8517e-07

{'Latency:Gain:TGSize' } {'Latency:Gain:TGSize' } {'std'} 7.8575e-11

{'invAmplitude:Gain:TGSize' } {'invAmplitude:Gain:TGSize' } {'std'} 5.8573e-05

{'Latency:invAmplitude:Gain:TGSize'} {'Latency:invAmplitude:Gain:TGSize'} {'std'} 1.0171e-06

Number of observations = 32353
Rsquared Adjusted = 0.8872
